# Congenital Transmesenteric Hernia in a Neonate

**Published:** 2015-01-10

**Authors:** Noora Al-Shahwani, Sheyma Al-Romaihi, Mansour J Ali, Parkash Mandhan

**Affiliations:** Department of Paediatric Surgery, Hamad General Hospital, Hamad Medical Corporation, Doha, Qatar

**Dear Sir**

Congenital transmesenteric hernia in neonates is a rare cause of intestinal obstruction with devastating outcomes. Patients are often managed with urgent surgical exploration and may require extensive bowel resection.[1-3] We present a case of a newborn presenting with intestinal obstruction found to have a strangulated transmesenteric hernia through a congenital defect in the ileal mesentery, which was managed successfully. 

A 5-hour-old newborn baby boy was admitted to neonatal intensive care unit for tachypnea and decreased activity. He was born at 39 weeks of gestation to a 33-year-old mother, with an unremarkable prenatal history. He was a product of an emergency cesarean section for antepartum bleeding. His Apgar score was 8 and 10 at 1 and 5 minutes respectively, with a weak cry. No active resuscitation was done. His birth weight was 3.2kg and growth performance was >50th percentile. On admission, baby had stable vital signs and mildly distended abdomen. His initial laboratory workup showed elevated white blood cell count and raised C-reactive protein. A plain abdominal X-ray at 8 hours of age showed a soft tissue shadow occupying right and mid abdomen with bowel pushed on left side with no free air in the peritoneal cavity (Fig.1). He was managed with nil per oral, orogastric tube, intravenous fluids and antibiotics. Within 12 hours of admission, he developed further abdominal distension associated with increased orogastric bilious output but remained clinically stable. At about 22 hours of age, the patient developed tachypnea and required elective intubation and mechanical ventilation. Further evaluation revealed distended and tense abdomen, increase in oro-gastric tube aspirate and change of aspirate color to dark greenish. The baby’s clinical findings prompted for early surgical intervention. 

**Figure:**
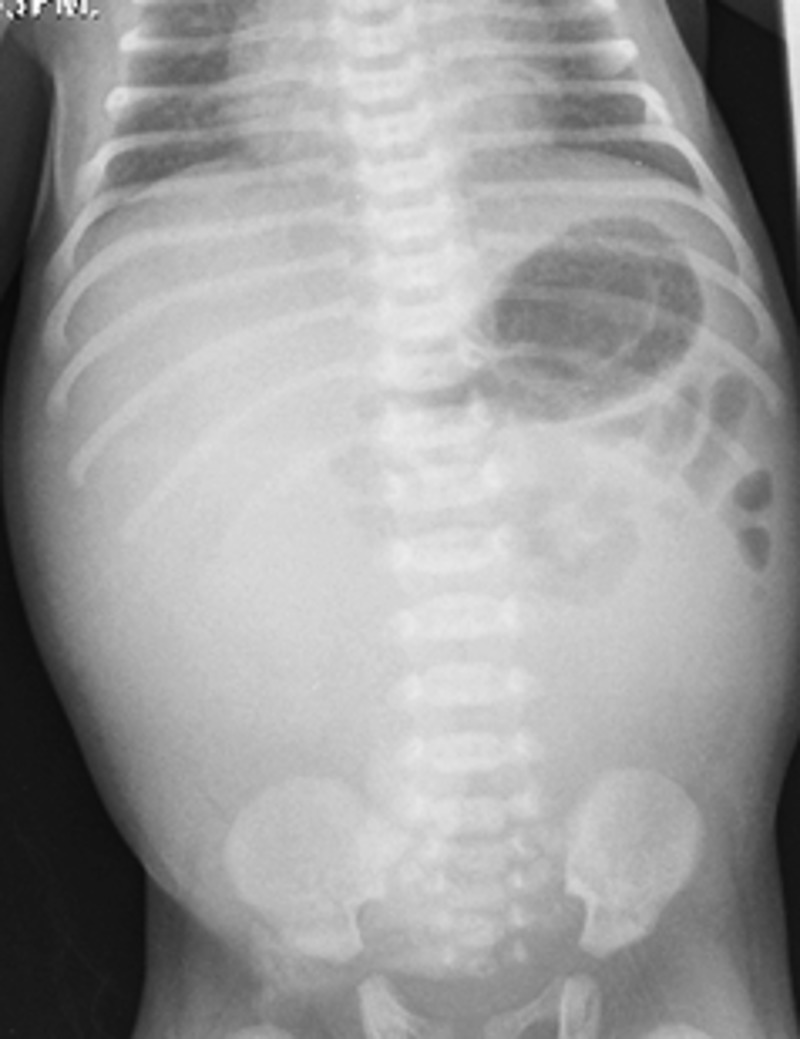
Figure 1: Plain X-ray of abdomen performed at 8 hrs. of age showing mass effect on the right side pushing bowel loops towards the left side with no free air in the peritoneal cavity.

Upon exploration, peritoneal cavity was full of meconium. A mesenteric defect was noticed at 30-cm from the terminal ileum (Fig. 2) through which a 15-cm segment of mid-ileum was herniated, which was ischemic and had multiple perforations. The gangrenous small bowel segment was resected and an end-to-end anastomosis was performed. The mesenteric defect was also repaired and after thorough wash of the peritoneal cavity, abdomen was closed. Patient’s post-operative course was smooth and he was started on feeds on 7th post-operative day. The histopathology of resected bowel confirmed infarcted small bowel. In follow up, patient has been reviewed in clinic at regular intervals and has remained stable with normal growth and development. 

**Figure:**
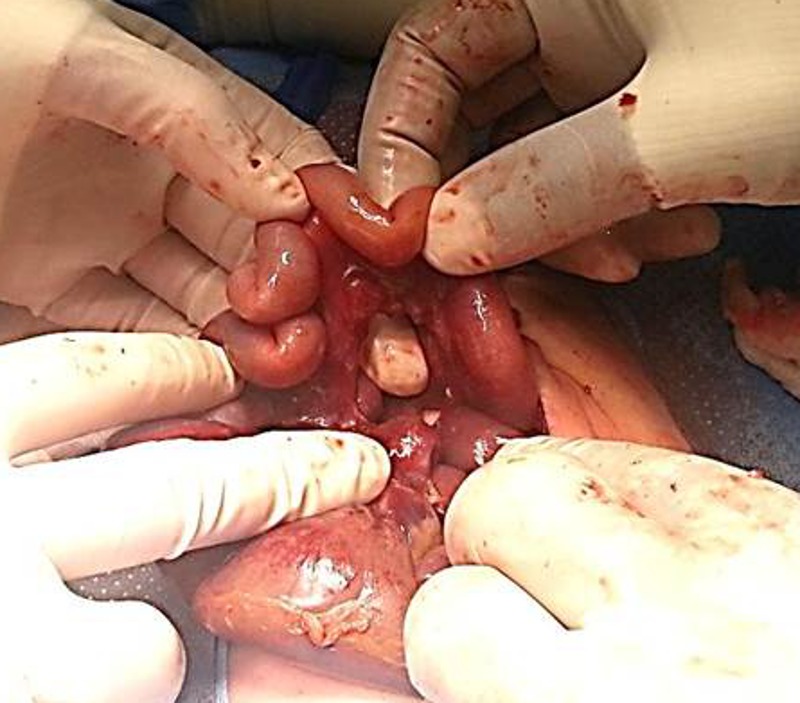
Figure 2: Intra operative finding after reduction of the hernia contents showing congenital mesenteric defect in the mid-ileum through which loops of small bowel herniated resulting in gangrenous bowel.

Congenital transmesenteric hernia (CTMH), a herniation of viscera through an anatomic defect of about 2-5 cm diameter in the mesentery, is rare in newborns. Less than 20 neonates with CTMH have been reported in English literature. Internal hernias are very rare and reported to occur in 0.2-0.9% of the populations in general, of which 5-10% are CTMHs [1,2,3] and at or near to the duodeno-jejunal junction [1].

CTMH in neonates often present as bowel obstruction [4-9], which may progress rapidly to strangulation and septic shock. Bowel ischemia has been reported as early as six hours after the onset of symptoms [1]. Murphy et al reviewed 11 pediatric cases with mesenteric hernias presenting with intestinal obstruction/strangulation, which included 9 neonates. Five of these neonates had associated bowel anomalies, like jejunal and ileal atresia, intestinal duplication, Hirschsprung’s disease, and malrotation [4]. In our case, the baby also presented within few hours after delivery with early signs and symptoms of small bowel obstruction but didn’t have any associated congenital malformations of gastrointestinal and other systems as contrary to Murphy et al cases.

In neonates, CTMH can be fatal and the outcome depends greatly on early diagnosis and intervention as the herniated bowel can rapidly progress to strangulation leading to sepsis and death. Janin and Murphy et al. have reported high morbidity and mortality in their series [1] [4]. In our case, even though we intervened early (within 11 hr. after surgical referral), the herniated bowel segment was infarcted with multiple perforations and peritonitis. After surgery, the patient recovered well. This highlights that an early diagnosis and surgical intervention is helpful to avoid the high morbidity and mortality in neonates with CTMH.

## Footnotes

**Source of Support:** Nil

**Conflict of Interest:** None

